# Selection of Representative Asphaltene Molecules in an Asphalt Molecular Model Based on Quantum Chemistry and Statistical Analysis

**DOI:** 10.3390/molecules29246015

**Published:** 2024-12-20

**Authors:** Jie Zhu, Ganyu Xia, Dejian Shen, Yangtao Li, Baosheng Jin, Shengxing Wu

**Affiliations:** 1 School of Architectural Engineering, Jinling Institute of Technology, No. 99, Hongjing Road, Nanjing 211169, China; 2 College of Civil and Transportation Engineering, Hohai University, No. 1, Xikang Road, Nanjing 210098, China; 3 College of Civil Engineering, Nanjing Forestry University, No. 159, Longpan Road, Nanjing 210037, China; 4 School of Energy and Environment, Southeast University, No. 2, Sipailou, Nanjing 210096, China

**Keywords:** asphalt molecular model, asphaltene, representative molecules, quantum chemical calculations, statistical analysis

## Abstract

Asphaltenes, as the most complex and strongly polar component among the four components of asphalt, have a significant impact on the macroscopic physicochemical properties of asphalt. Currently, the vast variety of molecular structures used to characterize asphaltenes increases the construction complexity of asphalt molecular models. To construct a more realistic asphalt molecular model and reduce the construction difficulty, this investigation obtains the molecular morphology, molecular polarity, and infrared spectrum indicators of 21 asphaltene molecules through quantum chemical calculations. Based on statistical analysis methods, the differences among asphaltene molecules are explored, and suggestions for selecting representative asphaltene molecules are proposed. The investigation shows that AS2, AS3, AS12, AS15, and AS17 are representative molecules that are significantly different from other asphaltene molecules. Among them, AS2, AS15, and AS17 are significantly different from the other molecules in terms of polarity and functional groups, while AS3 and AS12 are significantly different from the other molecules in terms of aromatic carbon percentage. This investigation is expected to provide valuable insights into the intrinsic relationship between the nanoscale characteristics and macroscopic properties of asphalt molecules.

## 1. Introduction

Asphalt is one of the most complex petroleum byproducts, widely used in road construction and coating industries. The macroscopic physical and chemical properties of asphalt, as well as the methods and effects of modification, have always been hot topics in asphalt research [1,2,3]. The macroscopic physicochemical properties of asphalt are typically determined by its nanoscale molecular structure. To investigate the properties of asphalt from a structural perspective, it is essential to construct a more realistic asphalt molecular model. Investigations into the molecular structure of asphalt date back to the 1960s, when Brown et al. [4] summarized the B-L method for calculating the elemental composition parameters of asphalt through nuclear magnetic resonance (NMR), elemental analysis, and vapor osmotic pressure methods. Lyu et al. [5] proposed an average molecular structure algorithm based on the B-L method which can automatically construct asphalt molecules based on NMR data, average molecular weight, and elemental composition of asphalt. Sheremata et al. [6] randomly combined aliphatic and aromatic molecules using Monte Carlo methods, selecting the molecule with the highest degree of agreement with the experimental results, creating the Sheremata method. Boek et al. [7] extended the Sheremata method to enable the generation of archipelagic asphaltene molecules. Through these methods, dozens of asphalt molecular models have been proposed which can be mainly divided into asphalt average molecular models and multicomponent asphalt molecular models.

In 1993, Jennings et al. [8] proposed eight asphalt average molecular models based on NMR test results. Cong et al. [9] utilized the SARA analysis method to determine the proportions of various elements and asphalt components in Liaoshu asphalt, construct an average asphalt molecular model, and implement it to investigate the compatibility of asphalt and modifiers at different temperatures. Sun et al. [10] constructed four average asphalt molecular models through elemental analysis, infrared spectrum, and NMR methods, and investigated the self-healing ability of asphalt based on these models. However, it is impossible to investigate the interactions between different asphalt components and the effects on macroscopic physicochemical properties due to the neglect of the differences between the various components of asphalt in the average asphalt molecular model. Therefore, multicomponent asphalt molecular models (three-component and four-component models) are proposed. Among them, the three-component model divides asphalt into asphaltene, resin, and oil, while the four-component model further divides asphalt into asphaltene, resin, aromatic, and saturate. Guo et al. [11] utilized the three-component model to explore the compatibility of asphalt molecules with rubber through molecular dynamic simulations. Zhu [12] determined the proportions of each asphalt component through four-component tests and constructed a four-component asphalt model utilizing the asphaltene molecular structure proposed by Dong et al. [13], the resin molecular structure proposed by Qi et al. [14], and the aromatic and saturate molecular structures proposed by Zhang et al. [15] and Storm et al. [16].

Among the components of asphalt, asphaltenes have the most complex structure, the strongest polarity, and are prone to precipitation, making them a current research hotspot. In terms of the molecular structure of asphaltenes, Yen et al. [17] proposed the continental asphaltene molecular model in 1961. Mullins et al. [18] improved the Yen model by considering the molecular weight and the number of aromatic rings in asphaltene molecules and proposed four Mullins models. Li et al. [19,20] pointed out the existence of the pentane effect in some asphaltene molecules and modified the Mullins models by altering the side chain positions, thus proposing the Li model. Additionally, various researchers have proposed different forms of asphaltene molecules based on asphalt from different sources. Storm et al. [16] conducted NMR experiments on asphaltenes from Latawi and Alaska’s North Slope and proposed the Storm model. Rogel [21] summarized the asphaltene molecular structure obtained by Carbognani through n-heptane precipitation and utilized it to investigate the solubility parameters of asphaltene monomers and aggregates. Artok et al. [22] separated asphaltenes from an Arabian crude oil mixture utilizing pentane and obtained the Artok model through methods such as pyrolysis gas chromatography and mass spectrometry, NMR, and gel permeation chromatography. Murgich et al. [23,24] extracted high-aromaticity asphaltenes from spectroscopic data of Venezuelan crude oil residues and proposed the Murgich model, using molecular dynamics to analyze the formation mechanism of micelles. Groenzin et al. [25] determined the size of asphaltene molecules by conducting fluorescence depolarization experiments and proposed the Groenzin model. Although the existing research has proposed many molecular structures in relation to asphaltenes, the different sources of asphalt typically lead to varying properties, making it difficult to accurately describe the properties of asphaltenes in various categories with a few asphaltene molecules. Factors such as the types of heteroatoms, the positions of functional groups, the size of aromatic rings, and the length of alkane chains in asphaltenes significantly affect the aggregation behavior and physicochemical properties. However, there is relatively little investigation into how to select asphaltene molecules, leading to arbitrary selection in the construction of asphalt molecular models which increases the deviation of simulation results from the actual performance.

To enhance the targeted selection of asphaltene molecules, it is necessary to investigate the differences among existing asphaltene molecules and select representative asphaltene molecules within the asphalt system. Researchers have employed statistical analysis methods such as principal component analysis and hierarchical clustering to preliminarily investigate the correlation between the macroscopic properties and the test indicators of asphalt. Margaritis et al. [26] measured the chemical and rheological properties of recycled asphalt and utilized hierarchical clustering to classify 19 asphalt samples into five groups, each representing different rheological properties and aging states. Liu et al. [27] analyzed the crack rate index of different asphalt pavement sections using ground-penetrating radar and classified the asphalt pavement sections through hierarchical clustering, effectively improving the division of maintenance sections. Siroma et al. [28] conducted a principal component analysis and hierarchical clustering on the phase angles of 23 asphalt types and evaluated their aging degree and cracking sensitivity. However, most current research focuses on exploring the macroscopic properties of asphalt through test indicators, with few researchers investigating the relationship between the nanoscale characteristics of asphaltenes and the selection of representative molecules. Quantum chemical (QC) calculations can provide nanoscale information such as charge distribution and infrared spectra of asphaltene molecules, facilitating a deeper understanding of the nanoscale properties. Therefore, it is feasible to utilize QC calculations to obtain the nanoscale properties of asphaltene molecules and investigate the selection of representative asphaltene molecules through statistical analysis methods.

In this investigation, we collect 21 asphaltene molecules (including most of the asphaltene molecules commonly used in asphaltene molecular modeling). QC calculations of asphaltene molecules are combined with principal component analysis and hierarchical clustering. A scheme is provided to quantitatively characterize the variability among asphaltene molecules. Based on this, we propose recommendations for the selection of representative molecules of asphaltene across different situations. The method developed through this investigation is characterized by good portability and could be applied to the study of other asphaltene component molecules. The results of this work are expected to reduce the complexity of constructing asphalt molecular models and provide valuable insights into elucidating the relationship between the nanoscale characteristics and macroscopic properties of asphalt molecules.

## 2. Calculation Method

**Figure 1:**
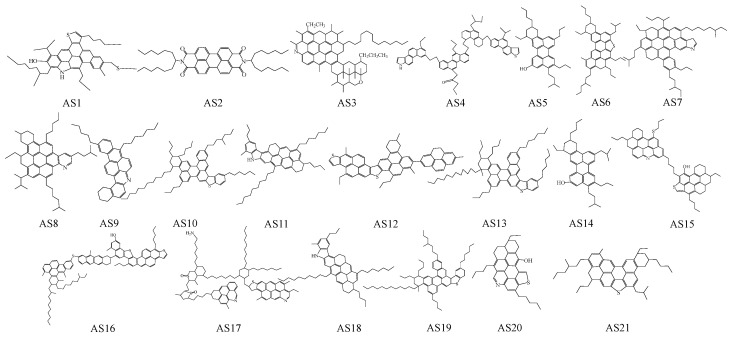
Molecular structure of asphaltenes.

This work collected a total of 21 asphaltene molecules (AS1–AS21) from various literature sources, as shown in Figure 1. Among them, AS1 represents the continental asphaltene molecular structure proposed by Mullins et al. [29], AS2 is the asphaltene molecular structure utilized by Nordgard et al. [30] to investigate the interfacial behavior of asphaltene oil-in-water emulsions, AS3 is the asphaltene molecular structure in the Lagunillas asphalt proposed by Yen [31], and AS4 is the archipelagic asphaltene molecular structure of the South China Sea crude oil proposed by Zhang et al. [32]. AS5–AS7 are modified asphaltene molecular structures proposed by Mullins [18], while AS8–AS11 are asphaltene molecular structures proposed by Yosadara [33]. AS12, AS16, and AS17 represent the insoluble asphaltene molecular structures of the Arabian crude oil mixtures proposed by Artok et al. [22]. AS13, AS18, and AS19 are ideal asphaltene molecular structures based on the number of aromatic carbons, alkane structures, and heteroatom content proposed by Groenzin et al. [25]. AS15 is the asphaltene molecular structure proposed by Takanohashi [34] after NMR analysis of asphaltene samples, and AS20 is the asphaltene molecular structure established by Wang et al. [35] through NMR analysis of Kuwait asphalt. AS14 and AS21 are modified asphaltene molecular structures proposed by Derek et al. [19,20] that take into account the pentane effect.

In this work, the molecular structures of asphaltenes were geometrically optimized utilizing Gaussian 09 [36] with the density functional theory. Specifically, the B3LYP functional was chosen for the calculations [37,38,39], and the 6–31 G(d, p) basis set was employed. Following optimization, vibrational frequency analysis was performed to ensure that all structures had positive vibrational frequencies, indicating local minima on the potential energy surface. The structures were subjected to wavefunction analysis by utilizing the Multiwfn 3.8(dev) software [40,41] to obtain relevant molecular descriptors. These descriptors could provide insights into the electronic and structural properties of the asphaltene molecules. Finally, the VMD 1.9.4 software [42,43] was utilized for visualization.

In this work, molecular weight, sp^2^ hybrid carbon atoms proportion, van der Waals volume, density, van der Waals surface area, span of deviation from plane (SDP), and molecular planarity parameters (MPP) were utilized to summarize the representative molecules with specific molecular morphology. The atomic dipole moment corrected Hirshfeld (ADCH) charge, the dipole moment, the MPI, the internal charge separation (Pi), the polar surface area ratio, the average Mayer bond order, and the HOMO-LUMO gap were utilized to summarize the representative molecules with specific molecular polarity. The Multiwfn software was utilized to calculate the infrared spectrum of asphaltene molecules, and the representative molecules with specific infrared spectra were summarized. Due to the difficulty of conducting infrared spectroscopic tests on specific asphaltene molecules and to the proven accuracy of Multiwfn in conducting molecular spectroscopic calculations, asphaltene infrared spectroscopic tests were not included in this study.

## 3. Results and Discussion

### 3.1. Molecular Morphology Indicators of Asphaltenes

This work analyzed the characteristics of 21 asphaltene molecules based on seven indicators: molecular weight, sp^2^ hybrid carbon atom proportion, van der Waals volume, van der Waals surface area, density, MPP, and SDP. The molecular morphology indicators for the asphaltenes are presented in Table 1.

According to Table 1, the molecular weights of asphaltenes varied widely, ranging from 525.77 g/mol to 1549.44 g/mol. Most of the sp^2^ hybrid carbon atoms in asphaltenes were distributed within the aromatic rings of the molecules, while a smaller portion was observed in C=O bonds. The sp^2^ hybrid carbon atom proportion in asphaltene molecules ranged from 30.26% to 77%, with a standard deviation of 10.75%, indicating significant differences in the proportions of saturated and aromatic carbons among different asphaltenes. The MPP values of asphaltenes ranged from 0.58 Å to 2.57 Å, while SDP values ranged from 2.82 Å to 13.93 Å. The density of asphaltene molecules ranged between 1.16 g/mol and 1.38 g/mol, indicating that the density difference among asphaltene molecules was not significant. The van der Waals volumes of asphaltenes exhibited a wide range of values, from 675.19 Å^3^ to 2095.69 Å^3^, and the van der Waals surface areas ranged from 562.35 Å^2^ to 1630.81 Å^2^, with standard deviations of 374.19 Å^3^ and 289.65 Å^2^, respectively. These results indicate significant differences in the van der Waals volumes and van der Waals surface areas among the different asphaltene molecules.

**Figure 2:**
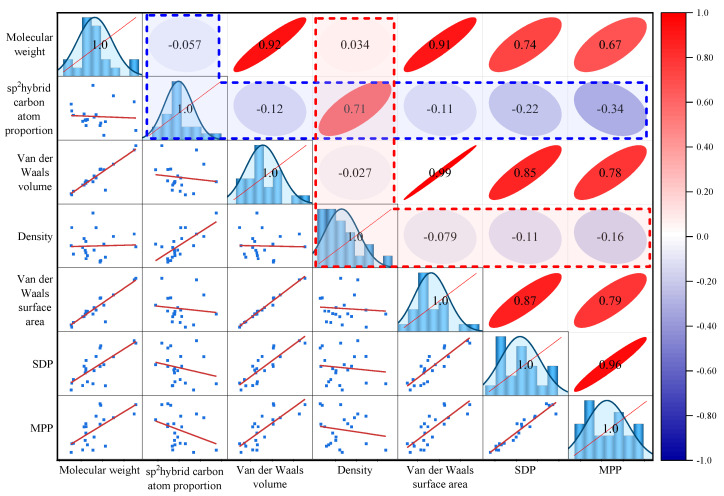
Correlation coefficients and scatter matrices of molecular morphology indicators of asphaltenes.

Considering the possibility of strong correlations among multiple molecular morphology indicators (such as those between van der Waals volume, van der Waals surface area, and molecular weight), this work conducted a correlation analysis by calculating the correlation coefficient between each pair of indicators, as shown in Figure 2.

As shown in Figure 2, the molecular morphology indicators of asphaltenes could be roughly divided into two categories. The first category included molecular weight, van der Waals volume, van der Waals surface area, SDP, and MPP, all of which exhibited strong positive correlations (correlation coefficients greater than 0.67). This was because an increase in the molecular weight of asphaltenes implies an increase in the number of atoms in the molecule, leading to a corresponding increase in the van der Waals volume and surface area. Additionally, the increased number of atoms also increases the probability of longer alkane chains in the molecule, a fact which reduced the planarity of the molecule and, subsequently, led to an increase in SDP and MPP. Among these five indicators, the correlations among van der Waals volume, van der Waals surface area, and molecular weight were strong (with pairwise correlation coefficients greater than 0.91). The correlation between MPP and SDP was closer (the correlation coefficient was 0.96), as MPP and SDP themselves are indicators to measure molecular planarity and are affected by factors such as the area of polycyclic aromatic hydrocarbon (PAH) regions in the molecule, as well as the length and direction of the alkane chain.

The second category of indicators included the sp^2^ hybrid carbon atom proportion and the density, as indicated by the red and blue dashed boxes in Figure 2. These two indicators showed low levels of correlation (absolute values below 0.34) and were negatively correlated with other indicators, while the correlation between them was relatively high (correlation coefficient was 0.71). This was because these two indicators reflect the proportion of various structures within asphaltene molecules. Asphaltene molecules typically consist of PAHs, alkane chains, aliphatic rings, and a very small number of heteroatoms. When the sp^2^ hybrid carbon atom proportion increased, the proportion of planar PAHs in asphaltene molecules increased, and the proportion of alkane chains that contributed more to SDP and MPP decreased. Such asphaltene molecules exhibited a more compact bonding pattern and a higher density.

The correlation analysis revealed a high degree of correlation among multiple indicators of asphaltene molecules. To reduce the adverse effects of these correlations on clustering, principal component analysis was performed on the molecular morphology indicators of the 21 asphaltene molecules after data standardization. The results are presented in Table 2.

According to Table 2, the eigenvalues of the first two principal components (*PC*1 and *PC*2) of asphaltene were both greater than 1.00, with variance percentages of 63.63% and 24.41%, respectively, resulting in a cumulative variance percentage of 88.04%. In contrast, the eigenvalues of the last five principal components were all lower than 1.00, with variance percentages below 6.31%. This indicates that *PC*1 and *PC*2 could capture the majority of the variation in the morphology indicators of asphaltene molecules. To investigate the specific manifestations of the differences among asphaltene molecules in terms of morphology indicators, scoring functions for each principal component were calculated. Among them, the results for the first two components are shown in Equation (1):

*PC*1 = 0.42*A* − 0.12*B* + 0.46*C* − 0.07*D* + 0.46*E* + 0.45*F* + 0.43*G*

**Figure 3:**
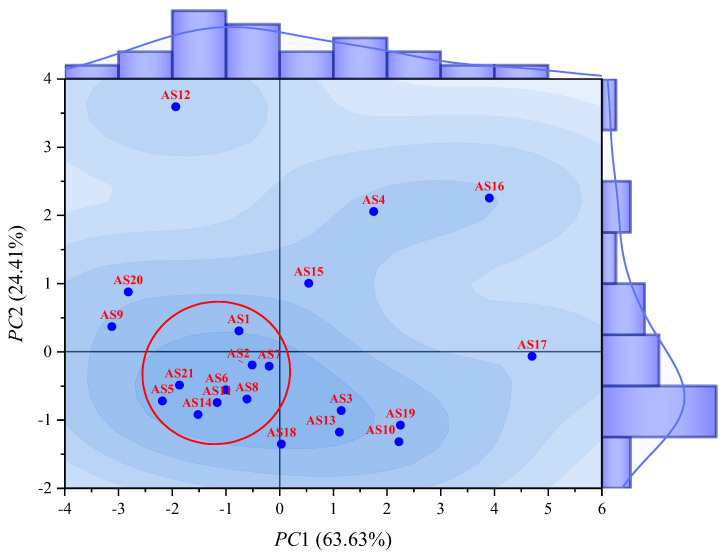
Map of principal component scores of asphaltene molecular morphology.

*PC*2 = 0.17*A* + 0.68*B* + 0.12*C* + 0.69*D* + 0.10*E* − 0.001*F* − 0.09*G* (1)

where *A*–*G* represent the standardized molecular weight, sp^2^ hybrid carbon atom proportion, van der Waals volume, density, van der Waals surface area, SDP, and MPP, respectively. In relation to *PC*1, the coefficients of *A*, *C*, *E*, *F*, and *G* were relatively large (with absolute values greater than 0.42), while the coefficients of *B* and *D* were smaller (with absolute values lower than 0.12). With respect to *PC*2, except for *B* and *D* which had larger coefficients (greater than 0.68), the absolute values of the coefficients of the other indicators were all lower than 0.17. The division of these two types of indicators was consistent with the results from the correlation analyses. It could be concluded that *PC*1 primarily reflected indicators representing the size of asphaltene molecules, such as molecular weight and van der Waals volume, while *PC*2 mainly reflected indicators representing the proportion of various internal structures within the molecule, such as the sp^2^ hybrid carbon atom proportion and density. The results of the principal component analysis were plotted as a principal component score plot, as shown in Figure 3.

**Figure 4:**
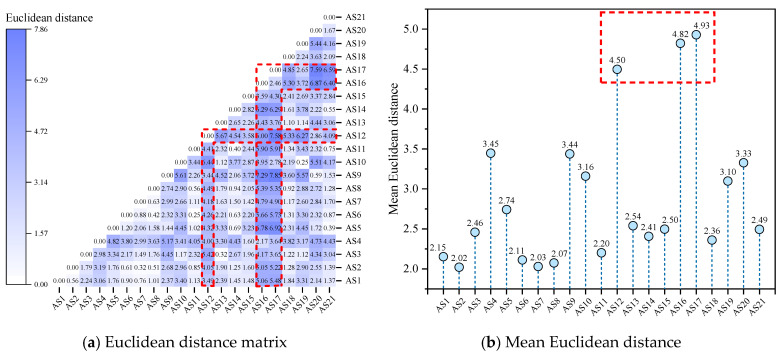
Euclidean distance based on molecular morphology indicators of asphaltenes.

As shown in Figure 3, the principal component score plot of asphaltene molecules exhibited a characteristic pattern of being denser on the left and bottom compared to the right and top. Within the range indicated by the red circle in Figure 3, there was a significant cluster of asphaltene molecules, including AS1, AS2, AS5–AS8, AS11, AS14, AS18, and AS21. These molecules were all continental asphaltenes with relatively small molecular weights and a proportion of sp^2^ hybrid carbon atoms ranging from 40% to 50%. In contrast, the remaining asphaltene molecules were more dispersed, particularly the archipelagic asphaltenes AS4, AS12, and AS15–AS17, all of which were located farther away from the others and exhibited distinct morphological characteristics compared to the other asphaltene molecules. This difference was attributed to the structural features of archipelagic asphaltenes. Continental asphaltenes were primarily composed of a single PAH plane surrounded by alkane chains, giving them a similar lamellar structure. In contrast, archipelagic asphaltenes contained multiple PAHs connected by flexible alkane chains or aliphatic rings. The varying relative positions of PAHs led to more diverse structural forms in archipelagic asphaltenes, resulting in large differences among different molecules. To better classify the 21 asphaltene molecules based on the molecular morphology, the Euclidean distance separating any two of them was calculated, and the results are shown in Figure 4.

**Figure 5:**
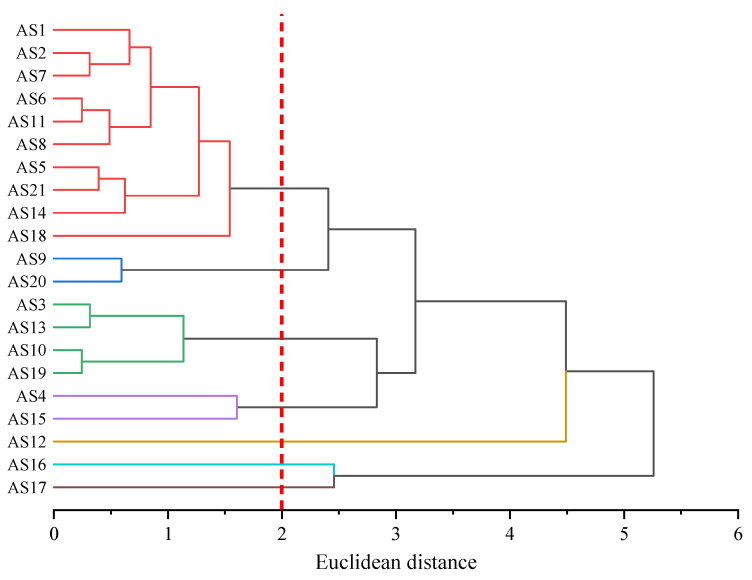
Hierarchical clustering based on morphology indicators of asphaltene molecules.

As shown in Figure 4, AS12, AS16, and AS17 were separated from other asphaltene molecules by larger Euclidean distances (mean values greater than 4.50). In the Euclidean distance matrix, the rows and columns where these asphaltene molecules are located are also darker in color (as shown in the red dashed box in Figure 4a), indicating significant differences in molecular morphology compared to other asphaltene molecules. Among them, AS17 exhibited the largest mean Euclidean distance from other asphaltene molecules (a distance of 4.93). Upon observation of the molecular structure of AS17, it could be concluded that AS17 was an archipelagic asphaltene molecule, and the alkane chain length between the two PAHs in AS17 was the longest (with 26 saturated carbon atoms in between), far exceeding other archipelagic asphaltene molecules (with the number of saturated carbon atoms in between being lower than or equal to 4). This means that AS17 had greater flexibility and a more variable structure, thus contributing to its larger Euclidean distance from other asphaltene molecules. Meanwhile, AS1, AS2, AS6—AS8, AS11, AS14, AS18, and AS21 were separated from other asphaltenes by smaller distances (mean distances lower than 2.5), exhibiting similar molecular morphological characteristics. Based on the Euclidean distances, a hierarchical clustering of asphaltene molecules was performed, and the results are shown in hierarchical Figure 5.

As shown in Figure 5, each of the 21 types of asphaltene molecules formed its own category when the distance was 0. As the distance increased, asphaltene molecules with similar molecular morphological characteristics began to merge into the same category. The larger the Euclidean distance at the time of merging, the greater the difference between the molecules. When the Euclidean distance was 2, the 21 types of asphaltene molecules were classified into seven categories according to their molecular morphology. Among them, there were more asphaltene molecules in the red part, except for AS18, all of which were molecules aggregated within the red circle in Figure 3. AS12, AS16, and AS17 molecules formed their own categories separately and were only classified when the Euclidean distance was greater than 2, indicating that they differed significantly in morphology from other molecules. Among these three asphaltene molecules, AS12 differed most significantly in terms of molecular morphology from other asphaltene molecules and was only classified into the same category as other asphaltene molecules when the Euclidean distance was close to 4.5. This was determined by the unique structural characteristics of AS12, which had the smallest molecular weight (871.20 g/mol) among the five archipelagic asphaltenes and the highest sp^2^ hybrid carbon atom proportion (76.19%) among the 21 types of asphaltenes. This resulted in significant differences in the scores of the two principal components of AS12 compared to all other asphaltene molecules. By summarizing the results of principal component analysis and hierarchical clustering analysis of molecular morphology indicators, it could be concluded that the differences in molecular morphology among asphaltene molecules were mainly reflected in the molecular weight and the sp^2^ hybrid carbon atom proportion. There were significant differences between archipelagic and continental asphaltene molecules, and there was also considerable diversity within archipelagic asphaltenes. AS12, AS16, and AS17 differed significantly in terms of molecular morphology from other asphaltene molecules.

### 3.2. Molecular Polarity Indicators of Asphaltenes

**Figure 6:**
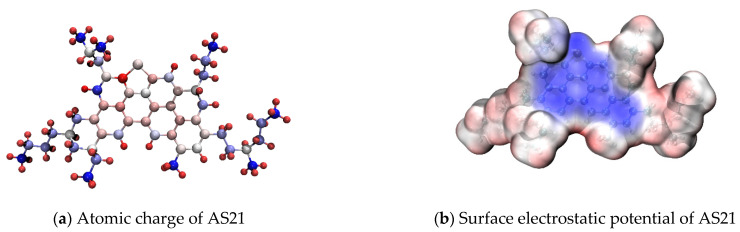
Atomic charge and electrostatic potential coloring of asphaltene molecules.

Asphaltene molecules have various polarity indicators, including dipole moment, atomic charge, and surface electrostatic potential. Taking AS21 as an example, its atomic charge coloring diagram and surface electrostatic potential coloring diagram are shown in Figure 6. The darker blue represents more negative charges or larger negative values of surface electrostatic potential, while the darker red represents more positive charges or larger positive values of surface electrostatic potential.

As shown in Figure 6, although both atomic charge and electrostatic potential coloring diagrams were indicators of molecular polarity, they exhibited distinct characteristics. In the atomic charge coloring diagram, PAHs appeared light in color, indicating a low charge carried by the atoms, while alkane chains and hydroxyl groups were darker, indicating a higher charge. Conversely, in the surface electrostatic potential coloring diagram, PAHs exhibited a deep blue color, while alkane chains were close to white, showcasing an opposite behavior. This difference arose due to the accumulation of delocalized π electrons on both sides of the PAH region which created a negative electrostatic potential without affecting the atomic charge value. Hence, PAHs appeared white in the atomic charge diagram. This observation underscores that a single polarity indicator cannot fully capture the polarity characteristics of asphaltenes, necessitating a combined analysis utilizing multiple polarity indicators.

**Figure 7:**
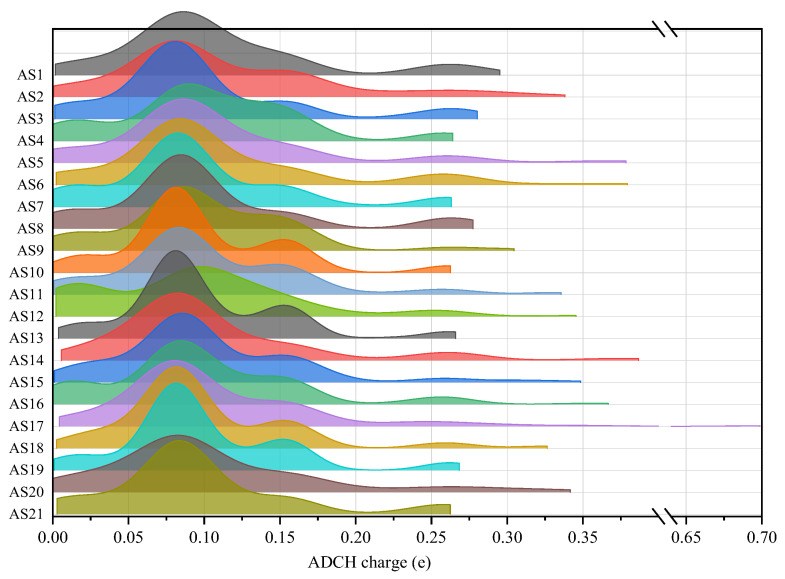
Distribution of the absolute ADCH charges of asphaltenes.

In this work, the polarity characteristics of asphaltenes were analyzed utilizing various indicators such as ADCH charges, the dipole moment, MPI, Pi, the polar surface area ratio, the average Mayer bond order, and the HOMO-LUMO gap of asphaltene molecules. To explore the differences in ADCH charges among asphaltene molecules, an analysis was conducted on 21 asphaltene molecules. To better reflect the dispersion of atomic charges within the asphaltene molecules, the absolute values of ADCH charges for each atom were taken and plotted as a distribution diagram, as shown in Figure 7.

According to Figure 7, the absolute ADCH charge distribution of most asphaltenes had two peaks. The highest peak was located around 0.08 e, with a relative frequency between 0.3 and 0.6; the lower peak was located around 0.15 e, with a relative frequency below 0.1. In addition, some atomic aggregations also appeared near 0 e and 0.26 e. Among them, the atoms with an absolute charge value of 0 e were mostly carbon atoms in PAHs, the atoms with an absolute charge value of 0.08 e were mostly hydrogen atoms, the atoms with an absolute charge value of 0.15 e were mostly saturated carbon atoms connected to two hydrogen atoms on the alkane chain, and the atoms with an absolute charge value above 0.30 e were mostly oxygen and nitrogen atoms. A comparison of 21 asphaltene molecules revealed differences in the distribution of atomic charges, particularly in terms of peak values and ranges. Notably, AS4 and AS12 exhibited lower peak values at 0.10 e (with relative frequencies below 0.3), attributed to their higher content of PAHs and relatively lower proportion of alkane chains. In contrast, AS5, AS6, AS14, AS16, and AS17 displayed a broader range of atomic charge distributions, with a maximum absolute value of atomic charges exceeding 0.35 e. Specifically, the maximum absolute value of atomic charges in AS5, AS14, and AS16 occurred on hydroxyl groups, while, in AS6, it was observed on a sulfur-substituted five-membered conjugated ring. Remarkably, the largest absolute value of atomic charge in AS17 was observed on the amino group (-NH_2_) at the end of the alkane chain, and its absolute value of atomic charge reached 0.70 e.

**Figure 8:**
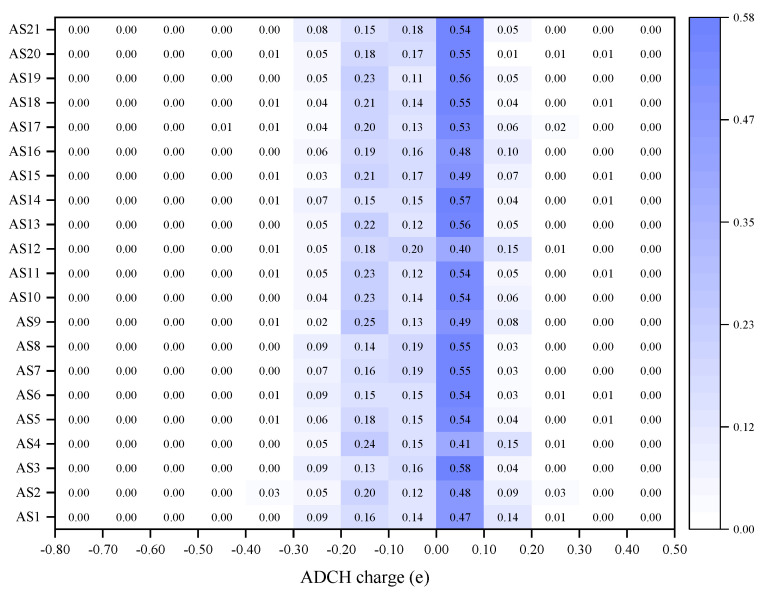
Thermogram of the ADCH charges of asphaltenes.

To evaluate the polarity of asphaltene molecules from the perspective of ADCH charges, the ADCH charge range (−0.80–0.50 e) was divided into 13 segments and the relative atomic frequencies of each asphaltene molecule in each segment were calculated, as shown in Figure 8.

As shown in Figure 8, the ADCH charge of most atoms in the asphaltene molecules was distributed in the range of −0.20–0.20 e, where the atomic relative frequency of 0.00–0.10 e was the largest, up to 0.5. Notably, AS4 and AS12 exhibited the lowest relative frequencies within the range of 0.00 e to 0.10 e, at 0.40 and 0.41 respectively, distinguishing them from other asphaltene molecules. A further principal component analysis was performed on the relative frequency of ADCH charges within each range for asphaltene molecules, and the results are shown in Table 3.

**Figure 9:**
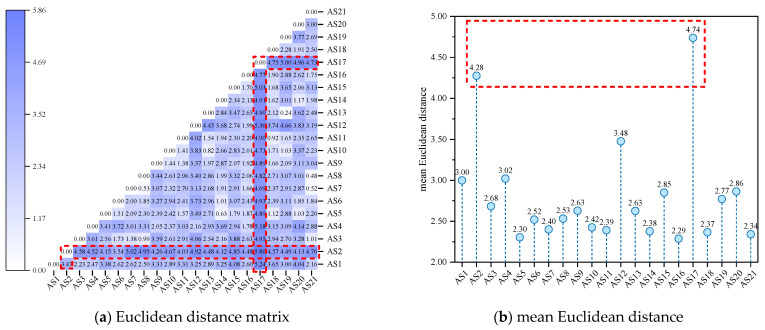
Euclidean distance based on the ADCH charges of asphaltenes.

As shown in Table 3, the cumulative variance percentage of the first four principal components reached 86.74%, which could reflect most of the differences in asphaltene molecules. The Euclidean distance between the asphaltene molecules was calculated according to the scores of the first four principal components of the ADCH charge, as shown in Figure 9.

**Figure 10:**
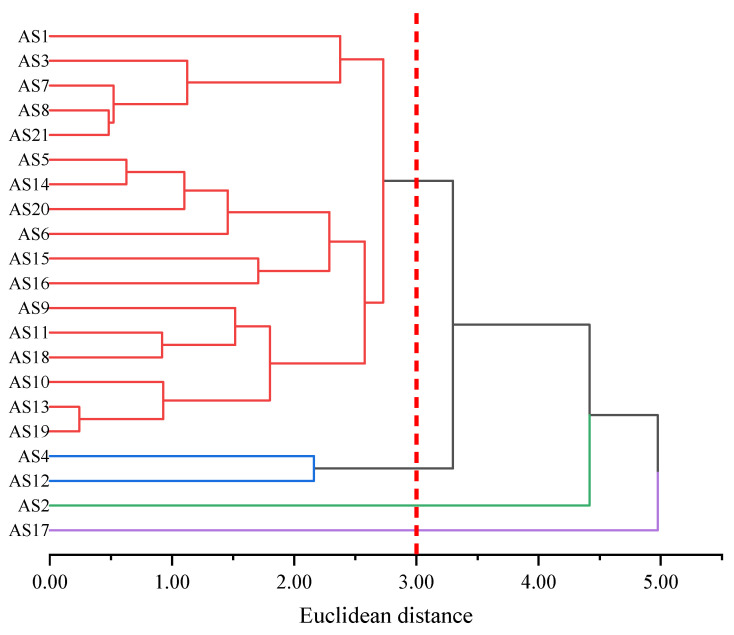
Hierarchical clustering of asphaltene molecules based on ADCH charges.

It can be observed that asphaltenes were divided into two categories based on the mean Euclidean distances from Figure 9b. One category included AS2 and AS17, which were separated from other asphaltenes by a larger mean Euclidean distance (greater than 4.00), indicating significant differences from most asphaltene molecules. In Figure 9a, it can be observed that the rows and columns where AS2 and AS17 are located are darker in color (as shown by the red dashed boxes), and the distances from these two molecules to other asphaltene molecules were relatively large (the distance between AS2 and other asphaltene molecules was greater than 3.47, and the distance between AS17 and other asphaltene molecules was greater than 4.69). This indicates that these two molecules had low similarity with any other molecules, exhibiting apparent specificity. The remaining asphaltene molecules belonged to the second category, which exhibited a smaller mean Euclidean distance (less than 3.50) from other asphaltene molecules, indicating that these asphaltene molecules had similar ADCH characteristics. Further, the 21 asphaltene molecules were clustered hierarchically in terms of ADCH charge, and the results are shown in Figure 10.

As shown in Figure 10, when the Euclidean distance was 3.00, asphaltene molecules were classified into four categories, with AS4 and AS12 belonging to the same category, a finding which is consistent with the analysis results in Figure 8. This was mainly due to the higher proportion of aromatic rings in AS4 and AS12, with the largest sp^2^ hybrid carbon atom proportion among all asphaltenes (66.67% and 76.19%, respectively). AS2 and AS17 each formed a separate category, and their first clustering was completed only when the Euclidean distance was greater than 4.00. It was observed that AS2 contained four carbonyl groups, while AS17 had an amino group and two sulfoxide groups which were absent in other asphaltene molecules. These strongly polar groups made the ADCH charges of AS2 and AS17 distinct from those of other asphaltene molecules.

In addition to the ADCH charge, this work also selected the dipole moment, MPI, Pi, polar surface area ratio, average Mayer bond order, and HOMO-LUMO gap to investigate the polarity characteristics of asphaltene molecules, as shown in Table 4.

**Figure 11:**
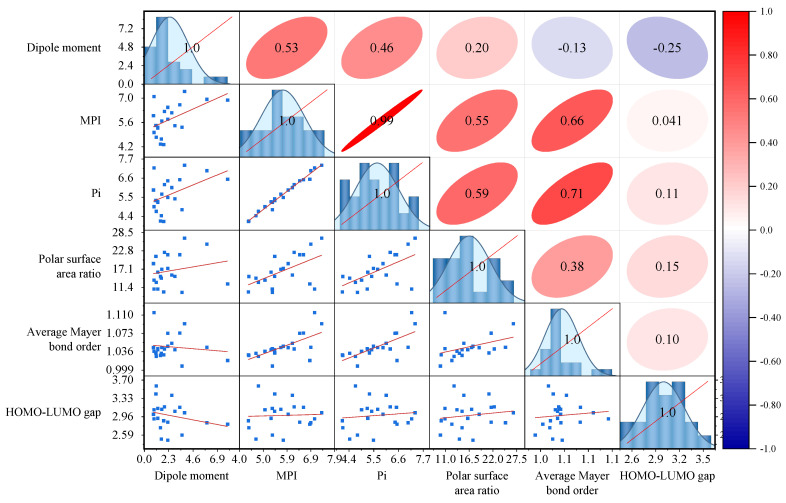
Correlation coefficients and scatter matrices of molecular polarity indicators of asphaltenes.

As shown in Table 4, the dipole moment of asphaltenes exhibited a wide distribution range (0.86–7.87 Debye) with a significant degree of dispersion (variance of 2.96 Debye^2^). This was because the dipole moment was not only related to the degree of charge distribution unevenness in asphaltenes, but also to their molecular morphology, with molecules having symmetric polar bonds tending to have smaller dipole moments. By comparing the MPI and Pi values of asphaltene molecules, it can be observed that the values were close. Specifically, the MPI ranged from 4.33 kcal/mol to 7.09 kcal/mol, with an average of 5.77 kcal/mol and a variance of 0.78 kcal/mol^2^, while the Pi ranged from 4.11 kcal/mol to 7.34 kcal/mol, with an average of 5.65 kcal/mol and a variance of 0.98 kcal/mol^2^. Considering that the correlation between different polarity indicators could affect the hierarchical clustering results, a correlation analysis of the polarity indicators of asphaltenes was performed, as shown in Figure 11.

As shown in Figure 11, there was a low correlation between dipole moments and the notational surface area share (correlation coefficient ≤ 0.2), and a moderate correlation between dipole moments and MPI and Pi, respectively (correlation coefficients ≥ 0.46). A significant positive correlation was found among MPI, Pi, and the polar surface area share (correlation coefficients ≥ 0.5). The average Mayer bond order of asphaltenes showed a significant positive correlation with MPI and Pi and a moderate correlation with the polar surface area ratio (correlation coefficient ≥ 0.38). This was because the high average Mayer bond order indicated the presence of more aromatic ring structures in asphaltene molecules which formed strong negative electrostatic potentials on both sides of the aromatic rings, leading to larger MPI, Pi, and polar surface area ratios. In contrast, the atomic charge difference between the alkane chain and the aromatic ring portions of asphaltene molecules was relatively small, resulting in a weaker correlation between the Mayer bond order and the dipole moment. The HOMO-LUMO gap showed weak correlations with other polarity indicators (absolute value of correlation coefficient ≤ 0.25), as it was more related to the reactivity of molecules rather than their polarity. This work further conducted a principal component analysis on the polarity indicators of asphaltene molecules and calculated the score functions of the first three principal components (*PC*1, *PC*2, and *PC*3), as shown in Equation (2).

*PC*1 = 0.26*H* + 0.55*I* + 0.55*J* + 0.39*K* + 0.41*L* + 0.06*M*

*PC*2 = −0.67*H* − 0.10*I* − 0.01*J* + 0.12*K* + 0.36*L* + 0.63*M*

**Figure 12:**
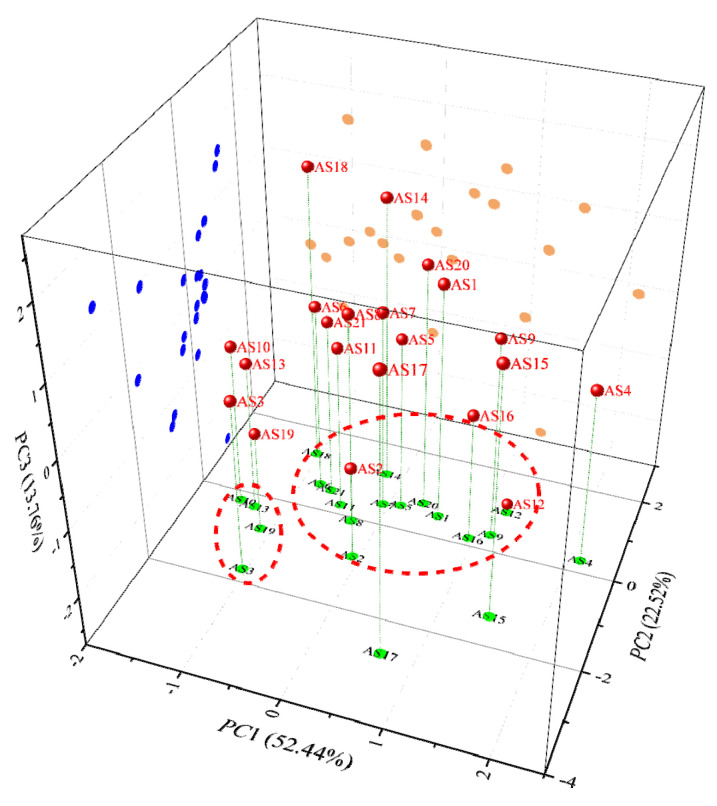
Map of principal component scores of asphaltene polarity indicators.

*PC*3 = 0.39*H* − 0.02*I* − 0.02*J* + 0.26*K* − 0.55*L* + 0.69*M* (2)

where *H*–*M* represent the dipole moment, MPI, Pi, polar surface area ratio, average Mayer bond order, and HOMO-LUMO gap of asphaltene molecules, respectively. In Equation (2), the coefficients of terms *I*, *J*, *K*, and *L* for *PC*1 were relatively large (with absolute values greater than 0.40), while those of *H* and *M* were smaller (with absolute values lower than 0.3). Notably, terms *I*, *J*, *K*, and *L* were all polarity indicators highly correlated with the surface electrostatic potential of asphaltene molecules, indicating that *PC*1 primarily reflected the electrostatic potential characteristics of the molecular surface. With respect to *PC2*, the coefficients of the terms *H* and *M* were larger (with absolute values greater than 0.60), while those of *I*, *J*, and *K* were smaller (with absolute values lower than 0.20), suggesting that *PC*2 mainly captured the dipole moment and the HOMO-LUMO gap characteristics of asphaltene molecules. Meanwhile, *PC*3 exhibited larger coefficients for terms *L* and *M* (with absolute values greater than 0.50) and smaller coefficients for *I*, *J*, and *K*, indicating that *PC*3 primarily reflected the HOMO-LUMO gap and bond order properties of asphaltene molecules. On this basis, a principal component score plot for each asphaltene molecule was generated, as shown in Figure 12.

**Figure 13:**
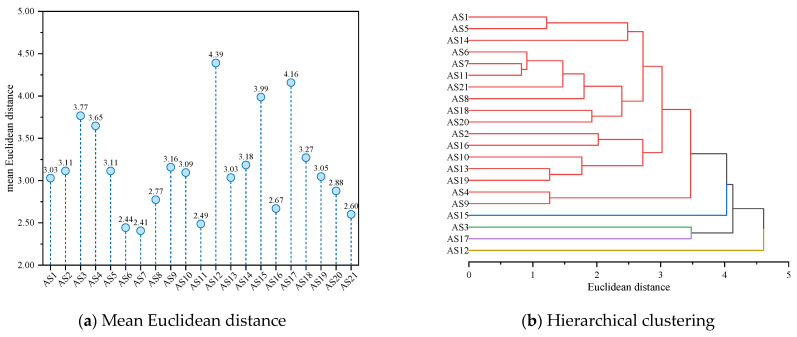
Mean Euclidean distance and hierarchical clustering based on the molecular polarity indicator of asphaltenes.

As shown in Figure 12, the three asphaltene molecules, AS4, AS15, and AS17, were relatively distant from the other asphaltene molecules, while the remaining 18 asphaltene molecules formed two clusters (as indicated by the red circles). Specifically, AS3, AS10, AS13, and AS19 were closer to each other and formed one cluster, while the remaining asphaltenes formed another cluster. To quantify the differences among the 21 asphaltene molecules, the mean Euclidean distance between any two of them was calculated, and a hierarchical clustering analysis diagram was plotted, as shown in Figure 13.

As shown in Figure 13b, AS3, AS12, AS15, and AS17 were gradually grouped together when the Euclidean distance exceeded 3.50, indicating that the polar characteristics of these molecules differed from those of other asphaltene molecules. This finding is corroborated by Figure 13a, which shows that the mean Euclidean distances separating these four asphaltene molecules (AS3, AS12, AS15, and AS17) from the other asphaltene molecules were also large, specifically 3.77, 4.39, 3.99, and 4.16, respectively. Among them, AS3 had the lowest average Mayer bond order and polar surface area ratio among the 21 asphaltene molecules (1.01% and 9.84%, respectively). Examination of its structure revealed a high proportion of saturated aliphatic rings and a relatively low proportion of aromatic rings which contributed to its low average bond order. In contrast, AS12 had the largest average Mayer bond order (1.11) and high MPI and Pi values (7.09 kcal/mol and 7.19 kcal/mol, respectively). The structure of AS12 showed a high proportion of aromatic rings which led to obvious polarity at the electrostatic potential level. At the atomic charge level, AS12 lacked oxygen and nitrogen atoms and had a high aromatic ring content, resulting in a relatively uniform distribution of atomic charges and a small dipole moment. AS15 ranked second in terms of dipole moment, MPI, Pi, and polar surface area ratio (5.92 Debye, 6.93 kcal/mol, 7.02 kcal/mol, and 24.92%, respectively). Its structure revealed an archipelagic asphaltene molecule containing a hydroxyl group and a pyrimidine, contributing to its relatively large dipole moment. From the structure of AS17, it could be observed that AS17 was an archipelagic asphaltene molecule. Unlike other asphaltenes, the amino group and sulfoxide group of AS17 made its polarity significantly different from other molecules (the sulfoxide group was generally considered to be the main oxidation product during the rapid aging period of asphalt, meaning that AS17 had some of the characteristics of aging asphaltenes). Specifically, the dipole moment of AS17 was significantly larger than that of other asphaltene molecules (that of AS17 was 7.87 Debye, the second highest was 5.92 Debye).

### 3.3. Infrared Spectrum Indicator of Asphaltenes

**Figure 14:**
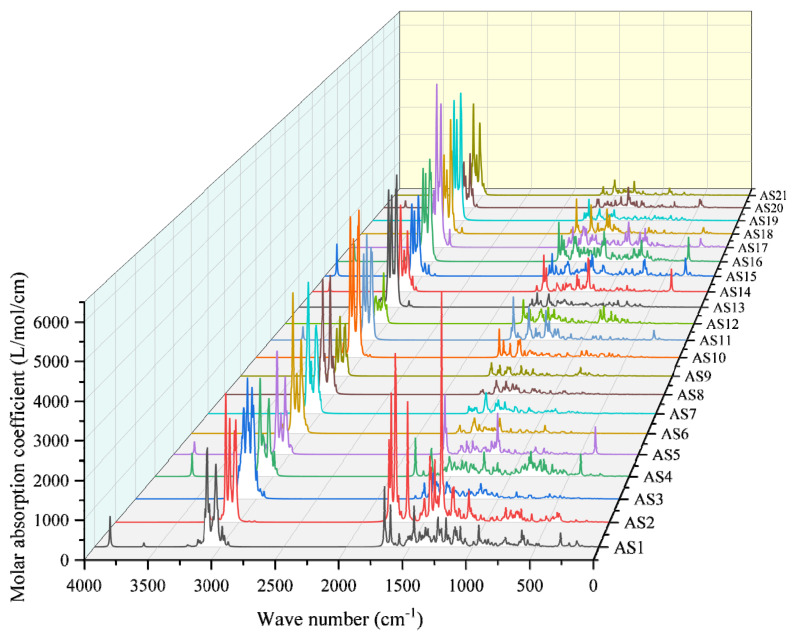
Infrared spectrum of asphaltenes.

Fourier infrared spectrum analyses typically play an important role in the process of determining the molecular composition of asphalt in traditional asphalt experiments. In infrared spectrum analyses, the infrared radiation of specific wavelengths is absorbed by chemical bonds of specific types and positions, so the structure of asphaltene molecules can be judged by their infrared spectrum. Since the infrared spectrum contains structural information on asphaltene molecules, it is helpful to describe the differences among asphaltene molecules. In this work, the infrared spectrum of asphaltene molecules was calculated and plotted, and the results are shown in Figure 14.

As shown in Figure 14, the absorption peaks of asphaltene molecules mainly appeared in two regions: one was near 3000 cm^−1^, while the other was within the range of 1750–500 cm^−1^, with most asphaltene molecules exhibiting higher peaks at 3000 cm^−1^. Additionally, some asphaltene molecules exhibited smaller absorption peaks at 3500–4000 cm^−1^ or 0–500 cm^−1^. Specifically, the peaks at 3000 cm^−1^ were primarily due to the stretching vibrations of C–H bonds, with those above 3120 cm^−1^ representing unsaturated C–H stretching vibrations, mostly corresponding to the stretching vibrations of C–H bonds at the edge of the aromatic rings in the asphaltene molecules. The absorption peaks within the range of 1750–500 cm^−1^ mainly involved three types of vibrations: bending vibrations of C–H bonds in alkanes, C=C skeletal vibrations in aromatic rings, and out-of-plane bending vibrations of C–H bonds at the edge of the aromatic rings. The peaks above 3500 cm^−1^ could be divided into two categories. The asphaltene molecules with absorption peaks within the range of 3750–3400 cm^−1^ included AS1, AS4, AS11, AS17, and AS18. These peaks represent the N–H stretching vibrations in these asphaltene molecules. Meanwhile, the molecules with absorption peaks within the range of 3750–4000 cm^−1^ included AS1, AS5, AS14–AS16, and AS20, and these peaks represent the O–H stretching vibrations of the hydroxyl groups in these asphaltene molecules. Furthermore, it was evident that the infrared spectrum of AS2 exhibited significant differences from that of other asphaltene molecules. Specifically, AS2 displayed several high peaks within the range of 1750–1250 cm^−1^, with a molar absorption coefficient reaching a maximum of 6012.78 L/mol/cm, which was higher than its peak near 3000 cm^−1^ and significantly higher than the peaks of other asphaltene molecules within this range. These high peaks correspond to the four ketone groups present in AS2.

**Figure 15:**
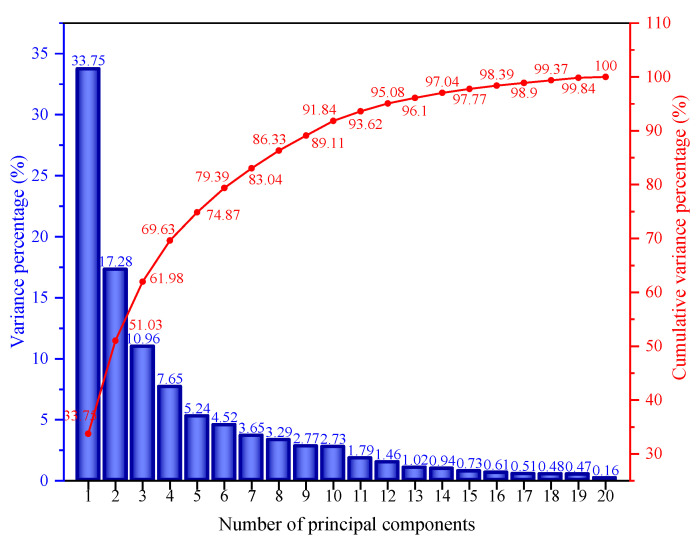
The variance percentage of the principal components of the infrared spectrum of the asphaltenes.

To quantify the differences among asphaltene molecules with respect to the infrared spectrum, a principal component analysis was performed on the infrared spectrum data of the asphaltene molecules, and the results are shown in Figure 15.

**Figure 16:**
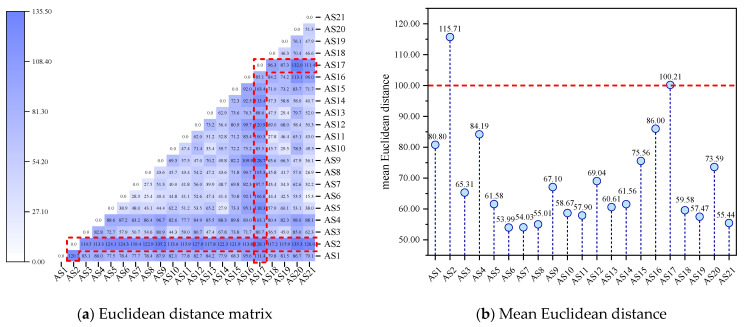
Euclidean distance based on the infrared spectrum data of the asphaltenes.

As shown in Figure 15, with the increase in the number of principal components, the corresponding variance percentage gradually decreased, and the increase in the cumulative variance percentage gradually decreased. This indicates that, as the number of principal components increased, the information on molecular differences contained in them gradually decreased. The work observed that the cumulative variance percentage of the first 20 principal components reached 100%, indicating that the first 20 principal components could summarize the differences among all asphaltene molecules. To quantitatively describe the differences among the asphaltene molecules with respect to the infrared spectrum, the Euclidean distance was calculated based on the first 20 principal components, and the results are shown in Figure 16.

**Figure 17:**
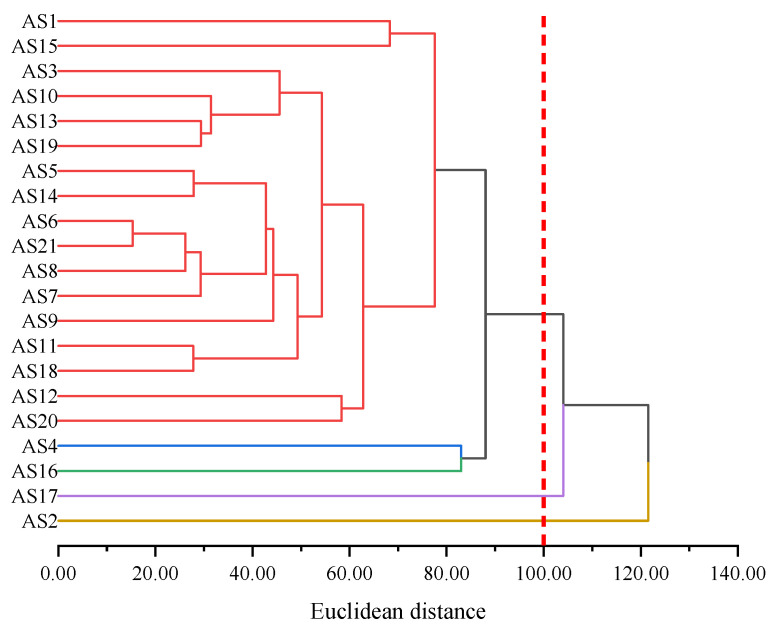
Hierarchical clustering of asphaltenes based on infrared spectrum indicators.

As shown in Figure 16a, the rows and columns where AS2 and AS17 are located are darker in color (as indicated by the red boxes), indicating that the infrared spectra of these two asphaltene molecules were significantly different from those of other asphaltene molecules. This conclusion is validated by Figure 16b: AS2 and AS17 had the largest mean Euclidean distances, both of which were above 100.00. To classify asphaltene molecules based on the infrared spectrum, this work conducted hierarchical clustering based on Euclidean distances, and the results are shown in Figure 17.

As shown in Figure 17, AS2 and AS17 gradually clustered when the Euclidean distance was greater than 100.00, indicating that AS2 and AS17 exhibited significant differences from other asphaltene molecules in terms of their infrared spectrum. By combining the infrared spectrum and structural characteristics of these asphaltene molecules, it can be observed that the difference in AS2 was mainly reflected in its four ketone groups, while the difference in AS17 was mainly reflected in its lower C/H ratio. Correspondingly, the peak value of the C–H stretching vibration band of AS17 was the highest among the 21 asphaltene molecules (exceeding 5500 L/mol/cm). At the same time, the unique polar functional groups (amino and sulfoxide) of AS17 also made its infrared spectrum significantly different from that of other molecules.

**Figure 18:**
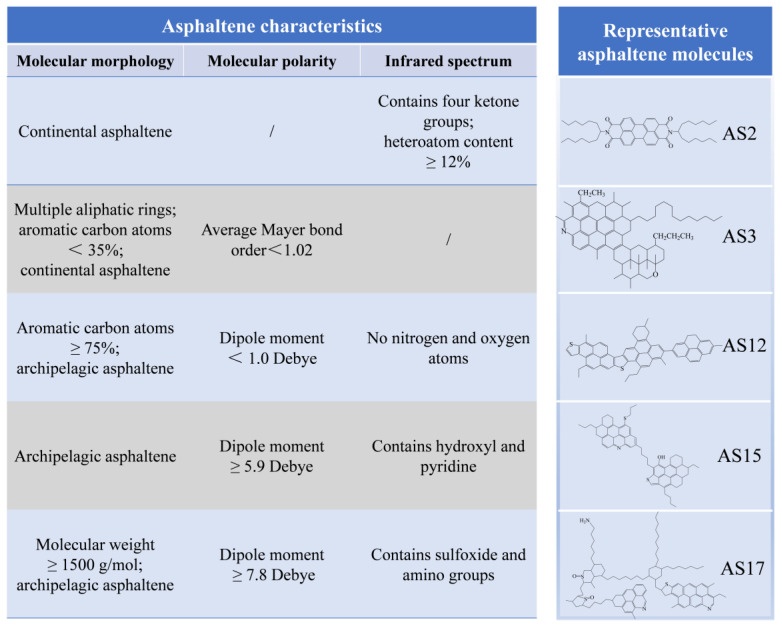
Recommendations for representative asphaltene molecule selection.

By comparing the molecular morphology, molecular polarity, and infrared spectrum analysis results of asphaltene molecules, it was observed that the clustering results of the asphaltene molecules under different indicators vary, but some molecules exhibited similar clustering properties under different indicators, such as AS12 and AS17. Among them, AS12 differed significantly from other asphaltene molecules in terms of molecular morphology, ADCH charge, and molecular polarity; AS17 exhibited significant differences from other asphaltene molecules in terms of hierarchical clustering analysis results under different indicators. By summarizing the analysis results under various indicators, specific molecules in the asphaltene could be identified which provided suggestions for the selection of representative asphaltene molecules in the construction of asphalt molecular models. Specific selection suggestions are shown in Figure 18.

As shown in Figure 18, if continental asphaltenes containing four ketones and over 12% of heteroatoms were to be selected, AS2 would be preferred. If continental asphaltenes containing multiple aliphatic rings and with fewer than 35% of aromatic carbons were to be selected, AS3 would be preferred. If archipelagic asphaltenes molecules with over 75% of aromatic rings, without nitrogen or oxygen atoms, and with a dipole moment lower than 1.0 Debye were to be selected, AS12 would be preferred. If archipelagic asphaltenes molecules with a dipole moment greater than 5.9 Debye were to be selected, AS15 or AS17 would be preferred; where AS15 contains a pyridine group, AS17 contains a sulfoxide group.

## 4. Conclusions

In this investigation, the molecular morphology, molecular polarity, and infrared spectrum indicators of 21 asphaltene molecules are obtained through QC calculations. Statistical analysis methods such as principal component analysis and hierarchical clustering are utilized to explore the differences among the asphaltene molecules, and specific molecules in the asphaltene molecules are summarized. Suggestions are provided for the selection of representative asphaltene molecules. The main conclusions are as follows.

(1) With respect to continental asphaltenes with four ketone groups and over 12% of heteroatoms, AS2 should be selected as the representative molecule.

(2) With respect to continental asphaltenes containing multiple aliphatic rings with fewer than 35% aromatic carbons, AS3 could be selected as the representative molecule. With respect to archipelagic asphaltenes with over 75% of aromatic rings, without nitrogen or oxygen atoms, and with a dipole moment lower than 1.0 Debye, AS12 should be selected as the representative molecule.

(3) With respect to archipelagic asphaltenes with a dipole moment greater than 5.9 Debye and containing hydroxyl groups and pyridine groups, AS15 could be selected as the representative molecule. With respect to archipelagic asphaltenes with a dipole moment greater than 5.9 Debye, a molecular weight greater than 1500 g/mol, and which contain sulfoxide groups and amino groups, AS17 should be selected as the representative molecule.

In future research, the experimental results of asphaltenes could be combined with quantum chemistry calculations to further explore the relationship between the macroscopic properties and microscopic structure of asphaltenes.

## Figures and Tables

**Table 1:** Molecular morphology indicators for asphaltenes.

Asphaltene	Molecular Weight (g/mol)	sp^2^ Hybrid Carbon Atom Proportion (%)	Van der Waals Volume (Å^3^)	Density (g/cm^3^)	Van der Waals Surface Area (Å^2^)	SDP (Å)	MPP (Å)
AS1	730.12	50.00	959.36	1.26	796.78	8.17	1.64
AS2	757.05	44.00	1004.90	1.25	853.37	7.36	1.77
AS3	1044.62	30.26	1399.58	1.24	1044.86	8.53	1.94
AS4	1194.69	66.67	1557.87	1.27	1267.97	10.31	2.02
AS5	560.85	48.78	779.49	1.19	662.55	5.17	0.96
AS6	749.18	44.44	1027.04	1.21	843.30	6.70	1.08
AS7	888.36	46.97	1216.95	1.21	992.10	7.20	1.22
AS8	758.17	44.64	1048.47	1.20	859.78	7.64	1.38
AS9	525.77	58.97	708.35	1.23	610.42	2.82	0.58
AS10	983.60	39.44	1408.70	1.16	1196.06	12.30	2.60
AS11	782.19	44.83	1060.17	1.19	909.19	4.68	0.90
AS12	871.20	76.19	1050.55	1.38	848.47	4.23	0.67
AS13	983.60	39.44	1405.39	1.16	1193.55	8.11	1.76
AS14	1068.60	42.86	811.42	1.18	678.89	3.78	0.84
AS15	920.36	49.21	1165.15	1.31	938.65	10.39	2.09
AS16	1524.26	61.11	1970.95	1.28	1595.24	13.93	2.18
AS17	1549.44	34.95	2095.69	1.23	1630.81	13.39	2.57
AS18	762.20	39.29	1077.41	1.17	913.79	9.50	1.65
AS19	995.61	41.67	1417.57	1.17	1201.66	12.78	2.54
AS20	533.77	52.78	675.19	1.31	562.35	4.36	0.94
AS21	707.10	43.14	958.74	1.22	792.40	3.84	0.68

**Table 2:** Principal component analysis of the molecular morphology indicators of asphaltenes.

Principal Component	Eigenvalue	Variance Percentage (%)	Cumulative Variance Percentage (%)
*PC*1	4.45	63.63	63.63
*PC*2	1.71	24.41	88.04
*PC*3	0.44	6.31	94.35
*PC*4	0.28	4.04	98.39
*PC*5	0.08	1.19	99.58
*PC*6	0.03	0.39	99.97
*PC*7	0.002	0.03	100.00

**Table 3:** Principal component analysis of ADCH charges of asphaltenes.

Principal Component	Eigenvalue	Variance Percentage (%)	Cumulative Variance Percentage (%)
*PC*1	3.10	31.03	31.03
*PC*2	2.21	22.06	53.10
*PC*3	1.89	18.86	71.95
*PC*4	1.48	14.79	86.74
*PC*5	0.78	7.80	94.53
*PC*6	0.41	4.06	98.59
*PC*7	0.11	1.12	99.72
*PC*8	0.03	0.28	100.00
*PC*9	0.00	0.00	100.00
*PC*10	0.00	0.00	100.00

**Table 4:** Molecular polarity indicators for asphaltenes.

Asphaltene	Dipole Moment (Debye)	MPI (kcal/mol)	Pi (kcal/mol)	Polar Surface Area Ratio (%)	Average Mayer Bond Order	HOMO-LUMO Gap (eV)
AS1	1.90	6.24	6.23	22.32	1.04	3.16
AS2	2.21	5.78	5.48	17.38	1.05	2.49
AS3	3.56	5.33	4.78	9.84	1.01	2.59
AS4	3.80	7.38	7.34	26.75	1.09	3.04
AS5	0.86	5.99	5.91	18.82	1.04	3.01
AS6	0.91	5.02	4.96	13.67	1.04	3.10
AS7	1.64	5.68	5.67	17.07	1.04	3.11
AS8	2.88	5.42	5.41	14.85	1.04	3.07
AS9	3.35	6.62	6.51	21.60	1.07	3.15
AS10	1.82	4.33	4.11	10.02	1.03	2.88
AS11	1.26	5.28	5.23	16.16	1.04	2.93
AS12	0.90	7.09	7.19	10.90	1.11	2.92
AS13	1.51	4.35	4.12	14.83	1.03	2.74
AS14	1.09	5.43	5.31	21.09	1.03	3.42
AS15	5.92	6.93	7.02	24.92	1.04	2.84
AS16	2.21	6.47	6.45	21.60	1.07	2.85
AS17	7.87	6.89	6.54	12.49	1.02	2.81
AS18	1.12	4.75	4.68	13.01	1.03	3.58
AS19	1.61	4.65	4.43	14.26	1.03	2.51
AS20	2.59	6.16	6.05	15.27	1.05	3.39
AS21	1.27	5.33	5.21	10.90	1.04	3.13

## Data Availability

The original contributions presented in this study are included in the article. Further inquiries can be directed to the corresponding author.
